# Supplementary material for: Bi3+ and Eu3+ Activated Luminescent Behaviors in Non-Stoichiometric LaO0.65F1.7 Structure
Source: Materials (Basel). 2020 May 19;13(10):2326. doi: 10.3390/ma13102326 (PMC7287885; doi:10.3390/ma13102326)
Supplement: Supplementary file 1 [file materials-13-02326-s001.pdf]

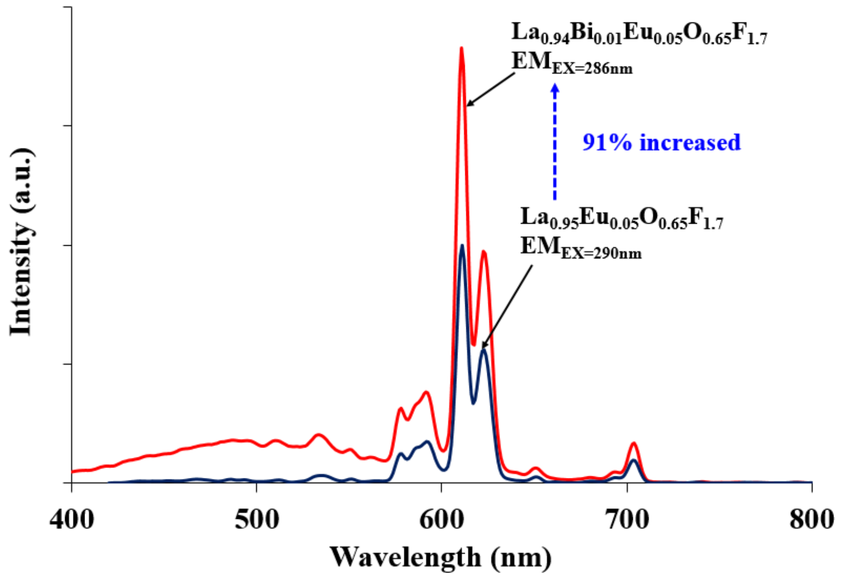

Figure S1. The integrated emission intensities of $La_{0.95}Eu_{0.05}O_{0.65}F_{1.7}$ and $La_{0.94}Bi_{0.05}O_{0.65}F_{1.7}$ phosphors.
